# Efficient Calculation of the Dispersion Energy for Multireference Systems with Cholesky Decomposition: Application to Excited-State Interactions

**DOI:** 10.1021/acs.jpclett.3c01568

**Published:** 2023-07-26

**Authors:** Michał Hapka, Agnieszka Krzemińska, Marcin Modrzejewski, Michał Przybytek, Katarzyna Pernal

**Affiliations:** † Faculty of Chemistry, University of Warsaw, ul. L. Pasteura 1, 02-093 Warsaw, Poland; ‡ Institute of Physics, Lodz University of Technology, ul. Wolczanska 217/221, 93-005 Lodz, Poland

## Abstract

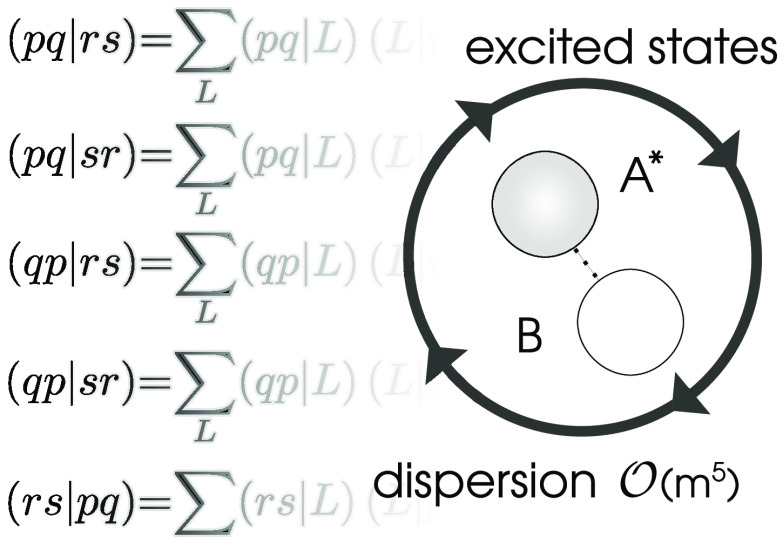

Accurate and efficient prediction of dispersion interactions in excited-state complexes poses a challenge due to the complex nature of electron correlation effects that need to be simultaneously considered. We propose an algorithm for computing the dispersion energy in nondegenerate ground- or excited-state complexes with arbitrary spin. The algorithm scales with the fifth power of the system size due to employing Cholesky decomposition of Coulomb integrals and a recently developed recursive formula for density response functions of the monomers. As a numerical illustration, we apply the new algorithm in the framework of multiconfigurational symmetry adapted perturbation theory, SAPT(MC), to study interactions in dimers with localized excitons. The SAPT(MC) analysis reveals that the dispersion energy may be the main force stabilizing excited-state dimers.

Modeling of dispersion forces is crucial for an accurate representation of noncovalent interactions in molecular systems^1,2^ and materials.^3−7^ Unfortunately, approaches to calculating the dispersion energy in excited-state complexes have been scarce.^8−13^ Semiempirical dispersion energy corrections for density functionals for ground-state complexes generally fail for dimers in excited states.^13,14^ So far, two pairwise dispersion approaches have been extended to excited states. First, the local response dispersion (LRD) model of Nakai and co-workers^8,15^ was applied to exciton-localized complexes from the S66 data set.^16,17^ Second, Feng et al.^18^ used the exchange-hole dipole moment (XDM) method of Becke and Johnson^19,20^ to obtain van der Waals *C*_6_ coefficients in systems involving inter- and intramolecular charge transfer excitations. The proposed generalizations of both LRD and XDM rely on the excited-state electron density extracted from TD-DFT.

When ground-state interactions are concerned, accurate values of the dispersion energy can be obtained from single reference symmetry-adapted perturbation theory (SAPT)^21,22^ based on either coupled-cluster^23−25^ or DFT description of the monomers.^26−29^ These methods are not applicable to excited states. Recently, we have developed a wave function-based approach to the dispersion energy in ground and excited states,^30,31^ which employs the extended random phase approximation (ERPA) for density response.^32^ The dispersion energy can then be predicted for any molecular system with a local exciton.^12^ A complete description of noncovalent interactions is accessible when combining our model with multiconfigurational SAPT,^33^ SAPT(MC), or a supermolecular approach based on the multiconfigurational self-consistent field (MCSCF), in particular, complete active space (CASSCF), description of the dimer.^34^ In the latter method, named CAS+DISP, the supermolecular CASSCF energy is corrected for the missing part of the dispersion energy. Both SAPT(MC) and CAS+DISP have already proven useful in studying excited-state organic dimers.^12^

Currently, the bottleneck in both SAPT(MC) and CAS+DISP is the calculation of coupled dispersion and exchange-dispersion energy contributions, as the computational cost of both components grows formally with the sixth power of the system size.

The goal of this work is to extend the applicability of the SAPT(MC) and CAS+DISP methods to larger systems by reducing the scaling of the coupled dispersion energy from *m*^6^ to *m*^5^. For this purpose, a novel algorithm is proposed. It employs a Cholesky decomposition technique and recently introduced recursive formula for the computation of density response functions.^35^ The *m*^5^ scaling is achievable if the interacting monomers are described with multiconfigurational (MC) wave functions, e.g., CASSCF, and the number of active orbitals is much smaller than that of virtual orbitals, which is typically the case. The new developments are applied to study molecular interactions in excited-state organic complexes of larger size than those affordable until now for multiconfigurational dispersion methods.

The approach for multireference functions parallels that in previous works focused on coupled dispersion energy computations for single-reference wave functions. In particular, SAPT based on the Kohn–Sham description of the monomers, SAPT(DFT),^28,29^ may employ either the density-fitting (DF)^36^ or Cholesky decomposition^37^ techniques, both leading to the *m*^5^ scaling of the dispersion energy computation. The algorithm of Bukowski et al.,^36^ recently improved by Xie et al.,^38^ is most general and applicable to computing the density response of the monomers from both local and hybrid functionals. In the case of the exchange-dispersion energy, the computational cost remains as large as *m*^5^ in single-reference SAPT (*m*^6^ in the multireference case)^31^ even in the DF/Cholesky formulation.^38−42^

The spin-summed second-order dispersion formula written in terms of monomer response properties obtained within the extended random phase approximation (ERPA) reads^30^
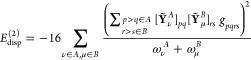
1where *pqrs* represents natural orbitals (NOs) of the monomers. Modified two-electron integrals in the NO representation are defined as
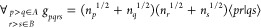
2where ⟨*pr*|*qs*⟩ represents two-electron Coulomb integrals in the ⟨12|12⟩ convention,  denotes a set of natural occupation numbers of monomer *X* (*X* = *A*, *B*), and it holds that 2*∑*_*p*∈*X*_*n*_*p*_ = *N*_*X*_, with *N*_*X*_ being the number of electrons in monomer *X*. (Occupation numbers of natural orbitals are, in general, fractional.) Transition energies, ω_ν_^*X*^, and transition vectors  follow from the ERPA equation^32,43^
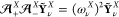
3where 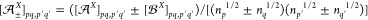 are Hessian matrices of the monomers (see ref (43)). The ERPA eigenproblem is expressed solely in terms of one- and two-electron spin-free reduced-density matrices. It should be noted that both the formula for the dispersion energy in eq 1 and the ERPA equation in eq 3 are applicable to closed- and open-shell systems with monomers in arbitrary spin states.

For multireference functions based on the partitioning of orbitals into the inactive (doubly occupied), active (partially occupied), and virtual (unoccupied) subsets, denoted as *s*_1_, *s*_2_, and *s*_3_, respectively, the range of the *pq* multi-index of  matrices, under the condition that *p* > *q*, can be split into the following subranges
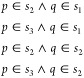
4The same partitioning also applies for the *p*′*q*′ multi-index. Thus, a straightforward implementation of ERPA requires steps that scale as *n*_OCC_^3^*n*_SEC_^3^, where  is the number of generalized occupied orbitals and  is the number of generalized secondary orbitals ( denotes cardinality of the set *s*_*i*_). An evaluation of the dispersion energy formula, eq 1, shares the same scaling behavior (indices μ and ν run over all *n*_OCC_*n*_SEC_ eigenvectors). Below we propose an algorithm leading to a lowered *m*^5^ scaling.

Using the integral identity
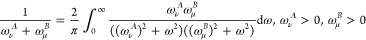
5and introducing the frequency-dependent matrix **C**^*X*^(ω)
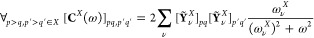
6leads to another representation of *E*_disp_^(2)^
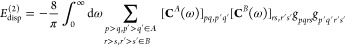
7The **C**^*X*^(ω) matrix is equivalent to the real part of the density linear response function taken with the imaginary argument i*ω* (see eq 33 in ref (44)) and is obtained by solving the following equation^35^
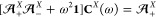
8

The modified two-electron integrals *g*_*pqrs*_ of eq 2 can be represented via the decomposition
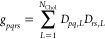
9where vectors **D**_*L*_ are obtained by Cholesky decomposition of the AO Coulomb matrix followed by transformation to the natural-orbital representation and scaling by *n*_*p*_^1/2^ + *n*_*q*_^1/2^ factors. The matrix product of **C**^*X*^(ω) with **D** yields a reduced-dimension intermediate
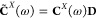
10which allows one to write
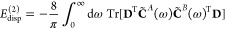
11Notice that the obtained formula applied to excited-state systems would miss the so-called non-Casimir–Polder terms arising from negative transitions ω_ν_^*X*^ < 0, i.e., transitions from higher- to lower-energy states, present in the density response function of an excited-state monomer. Extended RPA does yield negative excitations, which are considered spurious and discarded in practice, i.e., excluded from eq 1. In ref (12), we have shown how to account for negative excitations explicitly, in a physically meaningful manner. Upon inspection, we found that such non-Casimir–Polder terms are negligible for the studied systems, and they are not discussed any further.

The key step in the proposed reduced-scaling algorithm for computing dispersion energy with a multireference description of monomer wave functions assumes partitioning of a monomer Hamiltonian  into a partially correlated effective Hamiltonian, , and a complementary part,^30^. Then, the parametric representation of the Hamiltonian is introduced
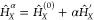
12where the  operator is multiplied by the coupling constant α ∈ [0, 1]. There are two underlying requirements in the Hamiltonian partitioning. The first one is that the wave function describing monomer *X* is of zeroth order in α for . The other condition is that scaling of the ERPA equations corresponding to  is lowered from *m*^6^ to *m*^5^ at α = 0. It has been shown that a group-product-function Hamiltonian^45,46^ satisfies both conditions for the MC wave function based on an ansatz assuming the partitioning of orbitals into inactive, active, and virtual ones. Notice that for a single-reference wave function,  can be chosen as a noninteracting Hamiltonian, as in the Møller–Plesset (MP) perturbation theory.

After employing the partitioned Hamiltonian  (eq 12), the resulting ERPA Hessian matrices  become linear functions of α. (Notice that from now on the index *X* in Hessian and response matrices is dropped for simplicity.)
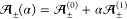
13By contrast, the response matrix **C**(α, ω) (eq 8) depends on α in a nonlinear fashion. Let us represent the projected response matrix **C̃**(α, ω) (eq 10) as a power series expansion in the coupling constant α around α = 0. Truncating the expansion at the *n*th order and setting α = 1, we obtain
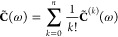
14where  follows from an efficient recursive scheme derived in ref (35)
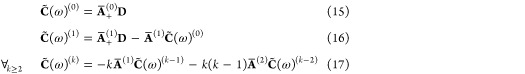
The required matrices are given by the ERPA matrices  and 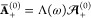
18
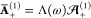
19
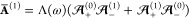
20
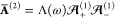
21with
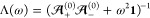
22Notice that by setting *n* = 0 in eq 14 for each monomer, the dispersion energy obtained from eq 11 will be equivalent to the uncoupled approximation introduced in ref (30). For a sufficiently large *n*, one recovers full dispersion energy, i.e., the coupled dispersion energy,^30^ numerically equal to that following from eqs 1–3.

Since the dimensions of the Hessian matrices and the matrix **D** are *m*^2^ × *m*^2^ and *m*^2^ × *N*_Chol_, respectively, matrix multiplications involved in eqs 15–17 scale as *m*^4^*N*_Chol_ ∼ *m*^5^. As has been shown in ref (46), the matrices  and  are block diagonal with the largest blocks of  size. Consequently, the cost of inversion of the Λ(ω) matrix (eq 22) is negligible if the number of active orbitals, , is much smaller than that of virtual orbitals, which is usually the case in practical calculations. The combination of eq 11 with the recursive scheme for  is the main contribution of this work.

A valid concern is whether the expansion of the linear response function at α = 0 (eq 14) leads to a convergent series. Although definite proof cannot be given, it is reasonable to expect that if a monomer wave function leads to stable ERPA equations around α = 0 then the series converges. Our numerical tests on two data sets of small, weakly correlated dimers^47,48^ have shown no convergence problems (Supporting Information). In most cases, expansion up to *n* = 8 was sufficient to achieve *μE*_*h*_ accuracy, amounting to the mean absolute percentage error below 0.1% in the dispersion energy. Convergence tests carried out on larger dimers, selected from the S66 test set, in both ground and excited states have led to the same conclusions; see Table S1 in the Supporting Information. An example of the convergence of *E*_disp_^(2)^ computed according to the procedure given by eqs 11–22 with the truncation order *n* in the range from *n* = 1 to 10 is presented for the benzene-cyclopentane complex in Figure S1 in the Supporting Information.

The evaluation of the α-expanded response matrix requires access to  Hessians together with three projected  matrices needed to carry out the recursion. Due to their size (*m*^2^ × *m*^2^ and *m*^2^ × *N*_Chol_, respectively), for systems approaching 100 atoms, these quantities can no longer fit into memory and have to be stored on disk. In this regime, the disk storage and the number of I/O operations will become the main bottleneck of the proposed approach.

The scaling of second-order induction energy computations in SAPT(MC) can be reduced to *m*^4^ using α-expansion of the response functions, accompanied by the Cholesky decomposition of two-electron integrals. However, it is possible to achieve such scaling without relying on the coupling constant expansion; see the Supporting Information for details. For the Hartree–Fock treatment of the monomers, such an alternative approach is identical to induction energy computations in the coupled Hartree–Fock scheme, as first proposed by Sadlej.^49^

Numerical demonstration of the developed algorithm was carried out for both ground and excited states of noncovalent complexes selected from the S66 benchmark data set of Hobza and co-workers.^16,17^ The dimers were divided into two sets according to their size. Smaller systems (up to eight heavy atoms in a dimer and ca. 500–600 basis functions with a basis set of a triple-ζ quality) were analyzed in detail in our recent work.^12^ Larger systems (up to 11 heavy atoms in a dimer and ca. 800–900 basis functions), which are beyond the capabilities of the *m*^6^-implementation of the MC dispersion energy, are analyzed for the first time. This group contains five complexes: benzene-cyclopentane, benzene-neopentane, AcOH-pentane, AcNH_2_-pentane, and peptide-pentane, where peptide refers to *N*-methylacetamide (see Figure 1).

**Figure 1:**
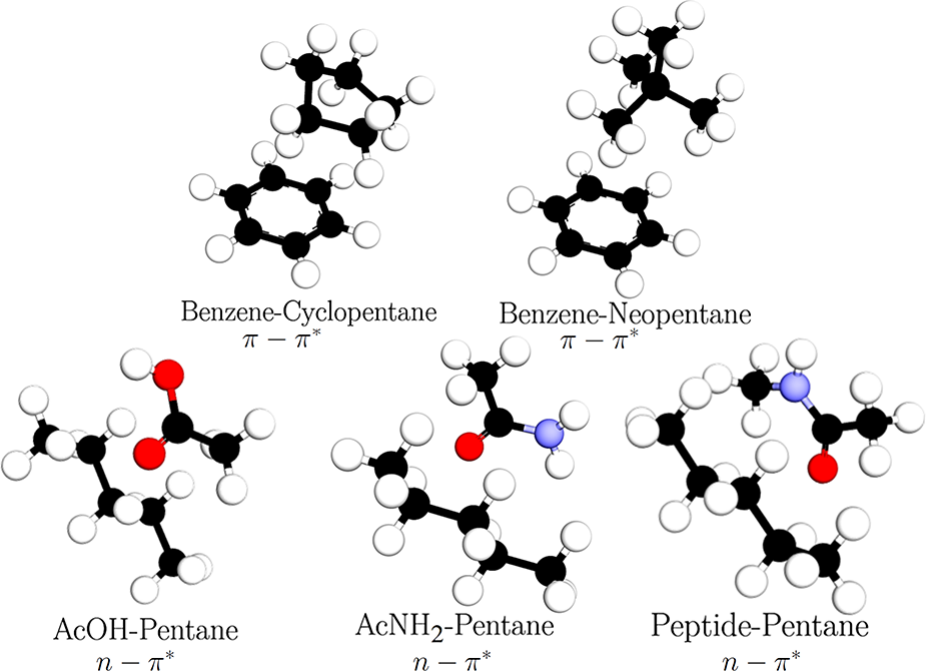
Structures of π–π* and n−π* complexes analyzed in this work.

Both ground- and excited-state calculations were performed using ground-state geometries taken from ref (16). All supermolecular calculations employed the Boys–Bernardi counterpoise correction.^50^ The excitons were localized on benzene (π → π*), AcOH (*n* → π*), AcNH_2_ (*n* → π*), and peptide (*n* → π*) molecules. The CCSD(T) results extrapolated to the complete basis set limit (CBS)^16^ served as a benchmark for ground-state interaction energies. In the case of complexes involving excited states, reference results were taken from ref (8). They were obtained by combining CCSD(T)/CBS ground-state interaction energies with excitation energies calculated at the EOM-CCSD^51^/6-31++G(d,p)^52−54^ level of theory.

The Cholesky decomposition of the Coulomb integrals matrix, ⟨*pr*|*qs*⟩, was performed in the AO basis with a modified program developed for refs (55) and (56). The Cholesky vectors, *R*_*pq*,*L*_, were generated with the convergence criterion ∑_*p*≥*q*_(⟨*pp*|*qq*⟩ – ∑_*L*_*R*_*pq*,*L*_*R*_*pq*,*L*_) < 10^–2^, which is the same as used in ref (35).

Second-order dispersion energies and SAPT(MC)^33^ energy components based on CASSCF treatment of the monomers were computed in the GammCor^57^ program. From now on, SAPT(MC) based on CASSCF wave functions is denoted as SAPT(CAS). The frequency integration in eq 11 has been carried out using the eight-point Gauss–Legendre quadrature. The necessary integrals and reduced density matrices were obtained from the locally modified Molpro^58^ package. Supermolecular CASSCF and DFT-SAPT calculations were performed in Molpro. All calculations employed the aug-cc-pVTZ basis set.^59,60^

Excited-state wave functions were computed with two-state-averaged CASSCF. We chose the same active spaces for both ground- and excited-state calculations. The active space for benzene included three π bonding and three π* antibonding MOs, which means six active electrons on six orbitals, labeled as CAS(6,6).^61^ For AcOH, we chose the CAS(8,8) active space including two n, π, π*, two σ, and two σ* orbitals.^62^ For AcNH_2_, the CAS(6,5) space was selected which involves σ, n, π, π*, and σ* orbitals.^63^ The peptide (*N*-methylacetamide) active space, CAS(6,6), was composed of σ, π, π*, and σ* orbitals, and two lone-pair orbitals *n* located on the oxygen atom.^64^

To improve the accuracy of the SAPT(CAS) interaction energy, especially for systems dominated by large polarization effects, we need to include higher than second-order induction terms. For ground-state systems which can be represented with a single Slater determinant, these terms can be approximated at the Hartree–Fock (HF) level of theory and represented as the δ_HF_ correction^65,66^
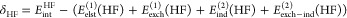
23where *E*_int_^HF^ corresponds to the supermolecular HF interaction energy and all terms are computed with Hartree–Fock wave functions. There is no straightforward way to account for higher-order polarization terms in excited-state computations. To tackle this problem, we assume that the change of higher-than-second-order induction terms upon excitation is proportional to a corresponding shift in the second-order induction and define the δ_CAS_ correction as
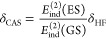
24where the labels GS/ES correspond to dimers in ground and excited states, respectively. Notice that in our previous work^12^ a similar scaling expression involved sums of induction and exchange-induction (*E*_exch–ind_^(2)^) terms. In this work, the latter is not computed directly but follows from an approximate scaling relation (see below). Such a treatment of *E*_exch–ind_^(2)^ energy could introduce additional error into the δ_CAS_ term, and we decided not to include it in eq 24.

Compared to single-reference SAPT schemes, in multiconfigurational SAPT it is not straightforward to apply the Cholesky decomposition to second-order exchange energy components, i.e., *E*_exch–ind_^(2)^ and *E*_exch–disp_^(2)^. The difficulty follows from the necessity to obtain separately lower and upper triangles of transition density matrices (see the discussion in section 2 of ref (31)); possible solutions will be addressed in our future work. To account for both exchange-induction and exchange-dispersion terms in this study, we propose a simple scaling scheme
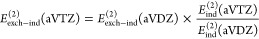
25
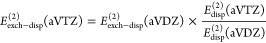
26where aVXZ = aug-cc-pVXZ, and it is assumed that the convergence of second-order polarization and exchange components with the basis set size is identical. Expressions for *E*_exch–ind_^(2)^ and *E*_exch–disp_^(2)^ terms are given in ref (33); the induction energy is computed with the *m*^5^-scaling algorithm presented in the Supporting Information.

All presented SAPT(CAS) results include δ_HF_ and δ_CAS_ corrections for ground- and excited-state complexes, respectively, as well as scaled second-order exchange components defined in eqs 25 and 26. CAS+DISP is a sum of the supermolecular CASSCF interaction energy and the dispersion energy, DISP = *E*_disp_^(2)^ + *E*_exch–disp_^(2)^, computed in the same fashion as in SAPT(CAS), i.e., using the newly developed expression given in eq 11 and the scaling relation from eq 26.

In Table 1, we present SAPT interaction energy decomposition for ground- and excited-state complexes. Regardless of the electronic state of the dimer, all systems can be classified as dispersion-dominated with the *E*_disp_^(2)^/*E*_elst_^(1)^ ratio ranging from 2.8 to 3.8.

**Table 1:** Components of the SAPT(CAS) Interaction Energy, Including the δ_HF/CAS_ Correction, for Ground- and Excited-State Complexes^a^

	*E*_elst_^(1)^	*E*_exch_^(1)^	*E*_ind_^(2)^	*E*_exch–ind_^(2)^	*E*_disp_^(2)^	*E*_exch–disp_^(2)^	δ_HF/CAS_	SAPT
	ground state							
Benzene-Cyclopentane	–2.05	5.33	–1.45	1.28	–7.13	0.87	–0.48	–3.63
Benzene-Neopentane	–1.58	4.08	–1.00	0.83	–5.58	0.65	–0.35	–2.95
AcOH-Pentane	–1.54	4.20	–1.05	0.83	–5.55	0.57	–0.28	–2.82
AcNH_2_-Pentane	–2.09	5.24	–1.57	1.01	–6.51	0.71	–0.39	–3.61
Peptide-Pentane	–2.31	6.07	–1.57	1.15	–8.03	0.85	–0.43	–4.26
	excited state							
Benzene-Cyclopentane	–1.92	5.15	–1.42	1.30	–6.89	0.82	–0.47	–3.42
Benzene-Neopentane	–1.43	3.87	–0.97	0.85	–5.38	0.61	–0.34	–2.80
AcOH-Pentane	–1.53	4.12	–1.03	0.91	–5.61	0.56	–0.27	–2.85
AcNH_2_-Pentane	–2.19	5.69	–2.05	2.01	–6.66	0.81	–0.52	–2.90
Peptide-Pentane	–2.27	6.11	–1.54	1.36	–8.10	0.88	–0.42	–3.97

	Δ*E*_elst_^(1)^	Δ*E*_exch_^(1)^	Δ*E*_ind_^(2)^	Δ*E*_exch–ind_^(2)^	Δ*E*_disp_^(2)^	Δ*E*_exch–disp_^(2)^	Δδ_HF/CAS_	SAPT
	excited state–ground state							
Benzene-Cyclopentane	0.14	–0.18	0.03	0.03	0.24	–0.05	0.01	0.22
Benzene-Neopentane	0.15	–0.21	0.03	0.02	0.20	–0.05	0.01	0.15
AcOH-Pentane	0.01	–0.08	0.02	0.08	–0.06	–0.01	0.02	–0.02
AcNH_2_-Pentane	–0.09	0.45	–0.48	1.00	–0.15	0.10	–0.12	0.71
Peptide-Pentane	0.04	0.04	0.03	0.21	–0.07	0.03	0.01	0.29

a The last column is a sum of all components. Differences in SAPT(CAS) energies between the excited (ES) and ground states (GS), Δ*E*_*x*_ = *E*_*x*_(ES) – *E*_*x*_(GS), are shown in the lower part of the table. Energy is given in kcal mol^–1^.

As can be deduced from Table 1, the most significant change in dispersion interactions upon transition from the ground to the excited state occurs in complexes of benzene (benzene···cyclopentane and benzene···neopentane). The effect amounts to Δ*E*_disp_^(2)^ ≈ 0.2 kcal mol^–1^, which corresponds to a decrease in the dispersion energy in the excited state. In both complexes, the redistribution of the electron density upon π → π* excitation on benzene is accompanied by a non-negligible drop in the electrostatic attraction. The latter energetic effect is, however, canceled by the simultaneous depletion of the exchange repulsion. Thus, a decline of the dispersion energy contributes in a major way to the weakened net attraction in the excited state. We observed the same trends in dimers of benzene with H_2_O, MeOH, and MeNH_2_ studied in ref (12).

Compared to benzene π → π* systems, complexes of *n*-pentane involve an *n* → π* exciton localized on the carbonyl group of the interacting partner (AcOH, AcNH_2_, and peptide). These systems exhibit an increase in the dispersion energy upon excitation, which ranges from −0.06 to −0.15 kcal mol^–1^ (Table 1). In AcOH···pentane, the enhanced dispersion is comparable in magnitude with a concurrent decrease in the first-order Pauli repulsion, both of which contribute to the overall stabilization of the excited-state dimer. In contrast, in peptide···pentane and AcNH_2_···pentane, the net repulsive components become stronger and outweigh the dispersion attraction so that both complexes are more strongly bound in the ground state. For peptide···pentane, the weakened interaction in the excited state can be attributed mainly to a significant increase in second-order exchange-induction (Δ*E*_exch–ind_^(2)^ = 0.21 kcal mol^–1^). The other interaction energy components undergo relatively minor changes; the only stabilizing effect of −0.07 kcal mol^–1^ is due to dispersion. In the case of AcNH_2_···pentane, increased static polarizability of acetamide in the excited state results in a stronger induction attraction. (The net change in induction and δ corrections amounts to −0.60 kcal mol^–1^.) This, however, is countered by a steep rise in the repulsive components. In particular, exchange-induction and first-order exchange increase by 1.00 and 0.45 kcal mol^–1^, respectively. Note that a similar pattern occurred in the methylamine···peptide (*n* – π*) interaction.^12^

The use of eq 11 enables spatial visualization of the dispersion interactions. Following the work of Parrish et al.,^67,68^ Wuttke et al.,^69^ and our recent development,^70^ we propose a spatially local dispersion density function , where *Q*^*X*^(**r**) are constructed from occupied natural orbital densities weighted by contributions to the dispersion energy. For details, see the Supporting Information. The *Q*^*AB*^(**r**) density function integrates to the dispersion energy
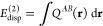
27The additional cost of the *Q*^*AB*^(**r**) computation is marginal, as all intermediate quantities are available from the calculation of *E*_disp_^(2)^.

Changes in the *E*_disp_^(2)^ components are visualized in Figure 2 by using the difference between the ground- and excited-state dispersion interaction density, *Q*^*AB*^(**r**). Both the sign and magnitude of the effect are correctly captured—one observes a notable depletion of the dispersion density in π – π* complexes compared to a weaker accumulation for *n* – π* dimers. In agreement with the character of the underlying excitons, in the π – π* case, the majority of the Δ*E*_disp_^(2)^ term is delocalized over the benzene ring, while in *n* – π* dimers it is basically confined to the carbonyl group.

**Figure 2:**
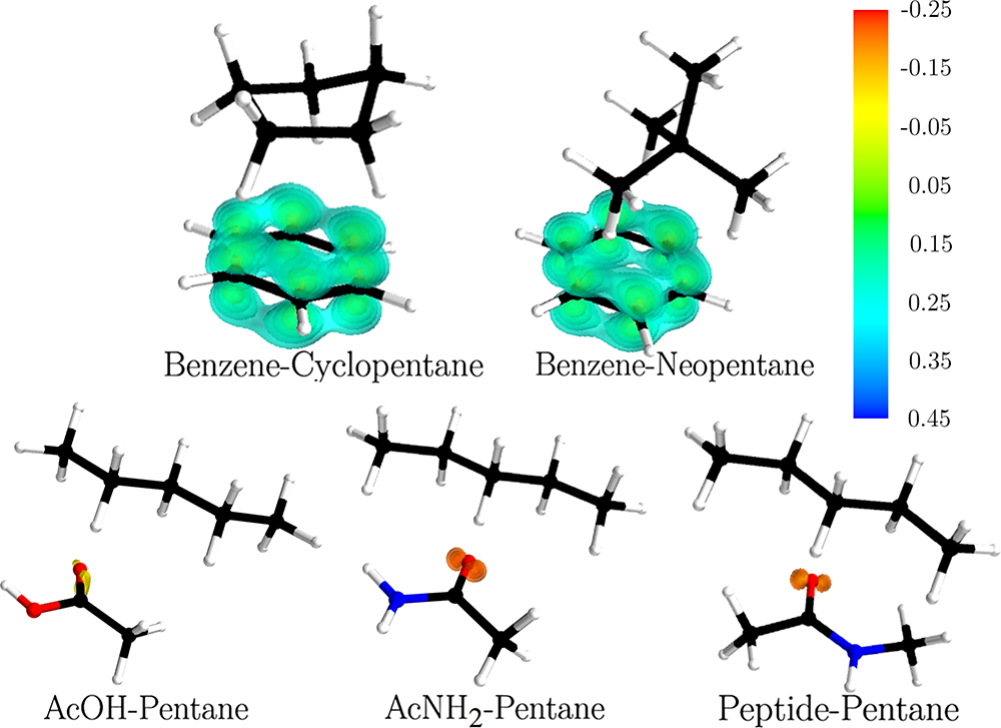
Differences in the dispersion energy density, *Q*^*AB*^(**r**), between excited and ground states. The presented isosurfaces encompass 50, 40, 30, 20, 10, and 1% of the integrated differences of *Q*^*AB*^(**r**) densities. Values on the color scale are reported in kcal mol^–1^ Å^–3^. Positive values correspond to regions where the dispersion energy density in the excited state is depleted (less negative) compared to the ground state.

In Tables 2 and 3, we report total ground- and excited-state interaction energies, respectively, calculated at the CASSCF, CAS+DISP, and SAPT(CAS) levels of theory. Addition of the dispersion energy to supermolecular CASSCF changes the character of the interaction from repulsive to attractive, reducing mean errors by 2 orders of magnitude. As a consequence, CAS+DISP results closely match the coupled-cluster (CC) reference^8^ with mean absolute percentage errors (MA%Es) of 0.8% for the ground and 3.7% for excited states. SAPT(CAS) performs similarly to the CAS+DISP model (MA%E values of 2.5 and 3.3% for ground and excited states, respectively). The DFT-based LRD model of Nakai et al.^8^ combined with the LC-BOP functional^71−73^ is somewhat less accurate. The model underestimates interaction energies, which amounts to mean errors at the level of 10% (Tables 2 and 3). Note, however, that the DFT results were obtained with the 6-311++G(2d,2p) basis set.

**Table 2:** Ground-State Interaction Energies,^a^ CCSD(T)/CBS Results from Reference (48), SAPT,^b^ TD-LC-BOP+LRD Results from Reference (8), and Mean Absolute Errors (MAE) and Mean Absolute Percent Errors (MA%E) Computed with Respect to the Reference

	CASSCF	CAS+DISP	SAPT	TD-LC-BOP+LRD	ref ^48^
Benzene-Cyclopentane	2.75	–3.51	–3.63	–3.04	–3.51
Benzene-Neopentane	2.06	–2.86	–2.95	–2.72	–2.85
AcOH-Pentane	2.18	–2.86	–2.82	–2.49	–2.91
AcNH_2_-Pentane	2.32	–3.48	–3.61	–2.96	–3.53
Peptide-Pentane	2.95	–4.23	–4.26	–3.47	–4.26
MAE	5.86	0.03	0.08	0.48	-
MA%E	172	0.8	2.5	13.4	-

a Given in kcal mol^–1^.

b Referring to SAPT(CAS) results, including the δ_HF_ correction.

**Table 3:** Interaction Energies in kcal mol^–1^ for π – π* (Benzene Complexes) and *n* – π* (Pentane Complexes) Excited States, in Which the SAPT Acronym Refers to SAPT(CAS) Results Including the δ_CAS_ Correction, EOM-CCSD(T) Values from Reference (8) Are Given as Reference, TD-LC-BOP+LRD Results Are Taken from Reference (8), and the Mean Absolute Errors (MAE) and Mean Absolute Percentage Errors (MA%E) Are Computed with Respect to the Reference

	CASSCF	CAS+DISP	SAPT	TD-LC-BOP+LRD	ref ^8^
Benzene-Cyclopentane	2.77	–3.31	–3.42	–3.11	–3.45
Benzene-Neopentane	2.06	–2.71	–2.80	–2.79	–2.86
AcOH-Pentane	2.27	–2.78	–2.85	–2.67	–3.03
AcNH_2_-Pentane	3.11	–2.74	–2.90	–2.48	–2.76
Peptide-Pentane	3.18	–4.05	–3.97	–3.52	–4.07
MAE	5.91	0.12	0.10	0.32	-
MA%E	184	3.7	3.3	9.6	-

Since the aug-cc-pVTZ basis set is not sufficient to saturate the dispersion energy with respect to the basis set size, the good agreement of both SAPT(CAS) and CAS+DISP with coupled cluster is partially due to error cancellation. (The CC values include CBS-extrapolated ground-state energies.) Indeed, individual SAPT(CAS) energy components for ground-state complexes are systematically underestimated with respect to their SAPT(DFT) counterparts (Tables S2–S4 in the Supporting Information). This reflects the effective neglect of intramonomer electron correlation effects in the SAPT(CAS) approach.^30,31,33^

To summarize, we have presented an algorithm for second-order dispersion energy calculations with multiconfigurational wave functions that scales with the fifth power of the system size. Until now, *m*^5^ scaling in coupled dispersion energy computations could be achieved only with a single-determinant description of the monomers.^29,36,38,39,74^ The prerequisite for *m*^5^ scaling with a multiconfigurational reference is that the number of active orbitals in wave functions of the monomers is considerably smaller compared to the number of virtual orbitals. In practice, this condition is typically fulfilled in interaction energy calculations performed using augmented basis sets.

The new *m*^5^ dispersion energy algorithm was employed together with state-averaged CASSCF wave functions to examine interactions involving localized excitons of the π – π* and *n* – π* types. For the representation of the interaction energy, multiconfigurational dispersion energy was complemented either with SAPT(MC)^33^ energy components or with supermolecular CASSCF interaction energy, the latter known as the CAS+DISP^34^ method. In line with earlier investigations,^12^ we have found out that in low-lying excited states the dispersion energy may be the driving force behind the stability of the complex. Hence, both accurate and efficient algorithms adequate for dispersion computations with multiconfigurational wave functions are mandatory. Spatial mapping of the dispersion energy density helps to identify the regions affected most by the exciton. Visualizing the remaining SAPT(MC) energy components could aid in the interpretation of energetic effects that occur upon electron density rearrangement in excited states. Work along this line is in progress.

## Data Availability

The raw data are available in the Zenodo repository at 10.5281/zenodo.8131535.

## Supporting Information Available

Description of the *m*^4^ algorithm for induction computations in the SAPT(MC) framework. Additional results including SAPT(DFT) and SAPT(CAS) energy decomposition, total CASSCF energies, and convergence of the dispersion energy with *n* for data sets in refs (16), (47), and (48) (PDF)
