# Supplementary material for: Efficient Calculation of the Dispersion Energy for Multireference Systems with Cholesky Decomposition: Application to Excited-State Interactions
Source: J Phys Chem Lett. 2023 Jul 26;14(30):6895–903. doi: 10.1021/acs.jpclett.3c01568 (PMC10405273; doi:10.1021/acs.jpclett.3c01568)
Supplement: Supplementary file 1 — jz3c01568_si_001.pdf [file jz3c01568_si_001.pdf]

Supporting Information: Efficient Calculation of Dispersion Energy for Multireference Systems with Cholesky Decomposition. Application to Excited-state Interactions

Michał Hapka,*,† Agnieszka Krzemińska,‡ Marcin Modrzejewski,† Michał Przybytek,† and Katarzyna Pernal‡

†*Faculty of Chemistry, University of Warsaw, ul. L. Pasteura 1, 02-093 Warsaw, Poland*

‡*Institute of Physics, Lodz University of Technology, ul. Wolczanska 217/221, 93-005 Lodz, Poland*

E-mail: michal.hapka@uw.edu.pl

Contents

1 Induction energy with multiconfigurational wave functions at the m^4 cost S-2

2 Visualisation of the dispersion energy S-4

3 Additional Results S-5

References S-12

1 Induction energy with multiconfigurational wave functions at the m^4 cost

Begin with the induction interaction energy expression introduced in Ref. S1

$$E^{(2)}_{\text{ind}} = E^{(2)}_{\text{ind}}(A \leftarrow B) + E^{(2)}_{\text{ind}}(B \leftarrow A) \quad , \tag{S1}$$

$$E^{(2)}_{\text{ind}}(A \leftarrow B) = -4 \sum_{\mu \in A} \frac{\left(\sum_{p>q \in A} \left[\tilde{\mathbf{Y}}^A_\mu \right]_{pq} \bar{\Omega}^B_{pq} \right)^2}{\omega^A_\mu} \quad , \tag{S2}$$

[$E^{(2)}_{\text{ind}}(B \leftarrow A)$ is defined analogously to $E^{(2)}_{\text{ind}}(A \leftarrow B)$], where $\bar{\Omega}^B$ is a vector of the modified interaction potential defined as

$$\forall_{p>q \in A} \quad \bar{\Omega}^B_{pq} = \left(n_p^{1/2} + n_q^{1/2} \right) \langle p | \hat{\Omega}_B | q \rangle \tag{S3}$$

$$\hat{\Omega}_B(\mathbf{r}) = v^B(\mathbf{r}) + \int \frac{\rho^B(\mathbf{r}')}{|\mathbf{r} - \mathbf{r}'|} \mathrm{d}\mathbf{r}' \quad . \tag{S4}$$

Equations are written in the natural orbitals representation of the monomer A and n_p, n_q denote natural occupation numbers, $\forall_p \; 0 \leq n_p \leq 1$. Using the expression for the frequency-dependent density response function [see Eq. (6) in the main text]

$$\left[\mathbf{C}^A(\omega) \right]_{pq,p'q'} = 2 \sum_\nu \left[\tilde{\mathbf{Y}}^A_\nu \right]_{pq} \left[\tilde{\mathbf{Y}}^A_\nu \right]_{p'q'} \frac{\omega^A_\nu}{\omega^2 + (\omega^A_\nu)^2} \quad , \tag{S5}$$

taken at $\omega = 0$ leads to

$$E^{(2)}_{\text{ind}}(A \leftarrow B) = -2 \left[\bar{\mathbf{C}}^A(0) \right]^{\mathrm{T}} \bar{\Omega}^B \quad , \tag{S6}$$

where

$$\forall_{p>q \in A} \quad \left[\bar{\mathbf{C}}^A(0) \right]_{pq} = \sum_{p'>q' \in A} [\mathbf{C}^A(0)]_{pq,p'q'} \bar{\Omega}^B_{p'q'} \tag{S7}$$

(analogous equations hold for the monomer B). The vector $\bar{\mathbf{C}}^A(0)$ can be easily found from Eq. (8) in the main text, written for $\omega = 0$ and projected on $\bar{\Omega}^B$,

$$\mathcal{A}_+\mathcal{A}_-\bar{\mathbf{C}}^A(0) = \mathcal{A}_+\bar{\Omega}^B \tag{S8}$$

where the $\mathcal{A}_+$, $\mathcal{A}_-$ are the hessian matrices constructed for the monomer A. Multiply Eq. (S8) by $[\mathcal{A}_+]^{-1}$ to obtain

$$\mathcal{A}_-\bar{\mathbf{C}}^A(0) = \bar{\Omega}^B \quad . \tag{S9}$$

Splitting $\mathcal{A}_-$ into zeroth- and first-order terms in the coupling constant α, see Eq. (13) in the main text taken for $\alpha = 1$,

$$\mathcal{A}_- = \mathcal{A}_-^{(0)} + \mathcal{A}_-^{(1)} \tag{S10}$$

leads to

$$\bar{\mathbf{C}}^A(0) = [\mathcal{A}_-^{(0)}]^{-1}\bar{\Omega}^B - [\mathcal{A}_-^{(0)}]^{-1}\mathcal{A}_-^{(1)}\bar{\mathbf{C}}^A(0) \quad , \tag{S11}$$

which gives rise to the iterative scheme

$$[\bar{\mathbf{C}}^A(0)]_{i+1} = [\bar{\mathbf{C}}^A(0)]_0 - [\mathcal{A}_-^{(0)}]^{-1}\mathcal{A}_-^{(1)}[\bar{\mathbf{C}}^A(0)]_i \quad , \tag{S12}$$

where

$$[\bar{\mathbf{C}}^A(0)]_0 = [\mathcal{A}_-^{(0)}]^{-1}\bar{\Omega}^B \quad . \tag{S13}$$

As it has been discussed in the main text, the matrix $\mathcal{A}_-^{(0)}$ is block diagonal with the largest blocks of the $M_{s_2}^2 \times M_{s_2}^2$ size, where M_{s_2} denotes the number of the active orbitals. Consequently, the cost of its inversion is negligible if the number of the active orbitals is much smaller than that of the virtual orbitals. All matrix multiplications in the proposed iterative are of the m^4 cost.

The iterative algorithm presented in Eq. (S12), has been implemented in GammCor using the DIIS accelaration technique.

2 Visualisation of the dispersion energy

By inspection, it can be checked that the dispersion energy expression given in Eq. (11) in the main text can be written in terms of a two-particle matrix $\mathbf{Q}$, indices of which correspond to occupied, i.e., inactive or active, orbitals localized on different monomers

$$E_{\mathrm{disp}}^{(2)} = \sum_{q \in A, s \in B} Q_{qs} \quad , \tag{S14}$$

where

$$\forall_{\substack{q \in A \\ s \in B}} \quad Q_{qs} = -\frac{8}{\pi} \int_0^\infty \mathrm{d}\omega \sum_{\substack{p \in A \\ r \in B}} \sum_{L=1}^{N_{\mathrm{Chol}}} D_{pq,L} W_{pq,rs}^{AB}(\omega) D_{rs,L} \quad , \tag{S15}$$

and

$$\forall_{\substack{pq \in A \\ rs \in B}} \quad W_{pq,rs}^{AB}(\omega) = \sum_{L=1}^{N_{\mathrm{Chol}}} \tilde{C}_{pq,L}^{A}(\omega) \tilde{C}_{rs,L}^{B}(\omega) \quad . \tag{S16}$$

Such a two-particle partition of the dispersion energy can be seen as a generalization of the partitioning scheme developed in Ref. S2 that was applied with uncoupled amplitudes and single-determinant wave functions.

We propose a local dispersion density function for monomer A as a charge-like density, where the density of the orbital is weighted by its contribution to the dispersion energy

$$Q^{A}(\mathbf{r}) = \sum_{q \in s_1^A \cup s_2^A} w_q \, \rho_q(\mathbf{r}) \quad , \tag{S17}$$

with weights defined as

$$\forall_{q \in s_1^A \cup s_2^A} \quad w_q = \frac{\sum_{s \in B} Q_{qs}}{N_q} \quad . \tag{S18}$$

$\rho_q(\mathbf{r})$ denotes either electron density of the active electrons if q refers to an active orbital localized on A

$$\forall_{q \in s_2^A} \quad \rho_q(\mathbf{r}) = \sum_{q' \in s_2^A} n_{q'} \, \varphi_{q'}(\mathbf{r})^2 \, , \qquad N_q = \sum_{q' \in s_2^A} n_{q'} \quad , \tag{S19}$$

(notice that the sum over active orbitals includes the orbital q) or an orbital density, if q

denotes an inactive orbital

$$\forall_{q\in s_1^A} \quad \rho_q(\mathbf{r}) = \varphi_q(\mathbf{r})^2 \ , \qquad N_q = 1 \quad . \tag{S20}$$

Analogous definition can be introduced by employing natural orbitals of the monomer B, leading to the dispersion density function localized on B, $Q^B(\mathbf{r})$. A function $Q^{AB}(\mathbf{r})$, defined as an average of $Q^A(\mathbf{r})$ and $Q^B(\mathbf{r})$,

$$Q^{AB}(\mathbf{r}) = \frac{1}{2}\left(Q^A(\mathbf{r}) + Q^B(\mathbf{r})\right) \quad , \tag{S21}$$

collects local contributions of the natural orbitals of both monomers to the dispersion interaction and, as it should, integrates to $E^{(2)}_{\text{disp}}$

$$E^{(2)}_{\text{disp}} = \int Q^{AB}(\mathbf{r})\ \text{d}\mathbf{r} \quad . \tag{S22}$$

The additional cost of obtaining the $Q^{AB}(\mathbf{r})$ descriptor is marginal compared to the cost of dispersion energy computation, as all intermediate quantities are available from the calculation of $E^{(2)}_{\text{disp}}$. Since natural orbitals are typically not localized, changing the $Q^{AB}(\mathbf{r})$ representation to local orbitals should provide a more informative visualization of dispersion forces. Our aim is, however, to investigate differential maps of $Q^{AB}(\mathbf{r})$ computed for ground and excited states of the studied systems. For this purpose, natural orbitals are adequate.

3 Additional Results

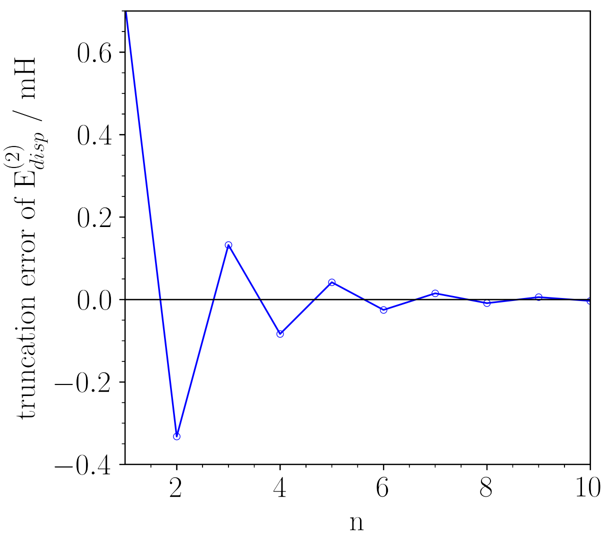

Figure S1: Convergence of the $E^{(2)}_{\mathrm{disp}}$ energy component as a function of the n-parameter, see Eqs. (11) and (14) in the main text, computed for benzen-cyclopentane complex in ground-state. Results obtained with aug-cc-pVTZ basis set.

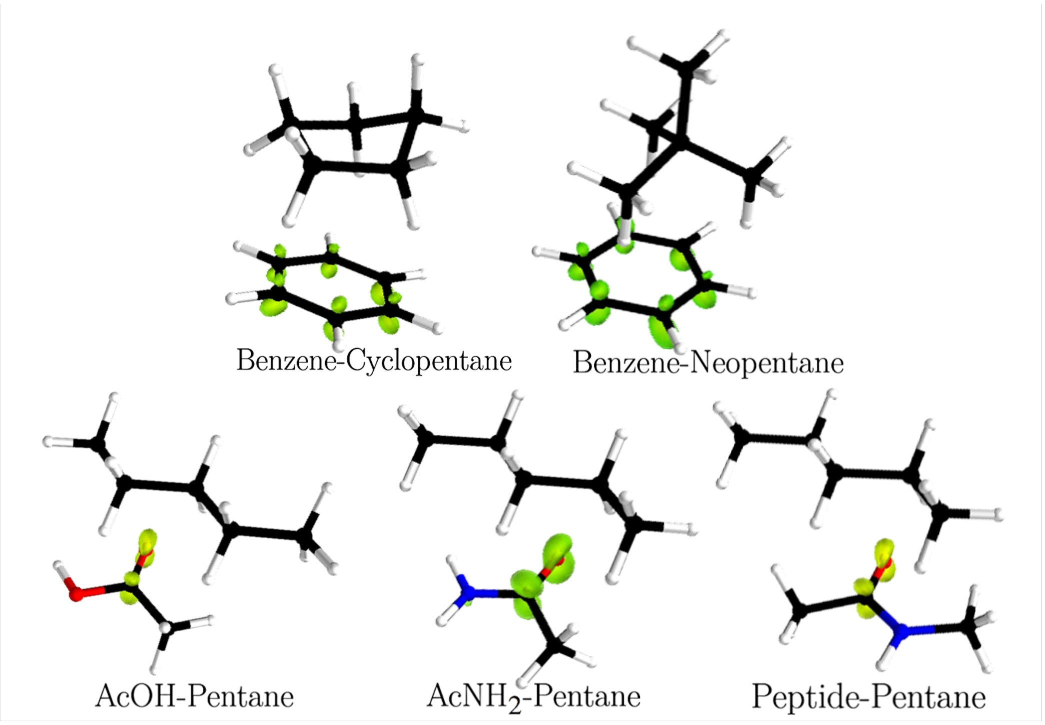

Figure S2: Differences of electron density between ground and $\pi \rightarrow \pi^*$ and $n \rightarrow \pi^*$ excited states respectively. The presented isosurfaces encompass 50%, 40%, 30%, 20%, 10% and 1% of the integrated differences of electron densities.

Table S1: Convergence of the dispersion energy with $n = 6, 8, 10, 12, 14$ with respect to the SAPT(CAS) reference (column labeled $E^{(2)}_{\text{disp}}$). The remaining columns present truncation errors, i.e., differences between the dispersion energy computed according to Eq. (1), shown in the main text, and the dispersion energy computed according to Eqs. (11) and (14), with monomers response functions truncated at different values of n. Basis set is aug-cc-pVTZ. Energy unit is μE_h.

ground state	6	8	10	12	14	$E^{(2)}_{\text{disp}}$
benzene-H_2O	−10.9	−4.0	−1.6	−0.6	−0.2	−4859
benzene-MeOH	−16.1	−6.0	−2.4	−1.0	−0.4	−7763
benzene-$MeNH_2$	−14.5	−5.2	−2.0	−0.8	−0.3	−7713
pyridine-H_2O	−29.7	−15.1	−8.0	−4.4	−2.4	−6499
pyridine-MeOH	−36.5	−18.6	−9.9	−5.4	−3.0	−7948
pyridine-$MeNH_2$	−26.3	−11.9	−5.9	−3.1	−1.7	−8217
peptide-H_2O	−8.3	−3.1	−1.2	−0.5	−0.2	−4652
peptide-$MeNH_2$	−9.4	−2.9	−0.9	−0.3	−0.1	−9057
MAE	19.0	8.4	4.0	2.0	1.0	
MAX	36.5	18.6	9.9	5.4	3.0	
excited state	6	8	10	12	14	ref.
benzene-H_2O	−6.7	−2.1	−0.7	−0.3	−0.1	−4589
benzene-MeOH	−9.4	−2.9	−1.0	−0.4	−0.1	−7377
benzene-$MeNH_2$	−8.0	−2.1	−0.6	−0.2	−0.1	−7367
pyridine-H_2O	−15.4	−5.8	−2.1	−0.6	−0.1	−6459
pyridine-MeOH	−18.9	−7.2	−2.6	−0.8	−0.2	−7891
pyridine-$MeNH_2$	−14.0	−4.3	−1.2	−0.2	0.1	−7978
peptide-H_2O	−8.0	−3.0	−1.2	−0.5	−0.2	−4674
peptide-$MeNH_2$	−9.2	−2.7	−0.7	−0.2	0.0	−9217
MAE	11.2	3.8	1.3	0.4	0.1	
MAX	18.9	7.2	2.6	0.8	0.2	

Table S2: **Ground-state** interaction energies (in kcal/mol) at the SAPT(DFT) and SAPT(CAS) levels of theory. "ref." denotes supermolecular CCSD(T)/CBS results of Ref. S3; aVXZ stands for aug-cc-pVXZ (X=D,T,Q) basis sets of Dunning. The SAPT/CBS values were obtained using a two-point aVTZ→aVTZ extrapolation scheme of Halkier et al.[S4] SAPT(DFT) calculations were performed with localized PBE0AC xc potentials for the monomers[S5] and the ALDA xc kernel. All SAPT values include the δ_{HF} correction.

	SAPT(DFT)			SAPT(CAS)		ref
	aVTZ	aVQZ	CBS	aVDZ	aVTZ	
benzen-cyclopentane	−3.35	−3.45	−3.52	−3.25	−3.63	−3.51
benzen-neopentane	−2.71	−2.78	−2.83	−2.66	−2.95	−2.85
AcOH-pentane	−2.61	−2.70	−2.77	−2.46	−2.82	−2.91
$AcNH_2$-pentane	−3.25	−3.36	−3.44	−3.15	−3.61	−3.53
peptide-pentane	−3.86	−3.98	−4.07	−3.79	−4.26	−4.26

Table S3: **Ground-state** interaction energy components (in milliHartree) at the **SAPT(DFT)** level of theory. aVXZ stands for aug-cc-pVXZ (X=T,Q) basis sets of Dunning. SAPT(DFT) calculations were performed with localized PBE0AC xc potentials for the monomers[S5] and the ALDA xc kernel. Second-order exchange energy terms given in the S^2 approximation.

	X	$E^{(1)}_{\mathrm{elst}}$	$E^{(1)}_{\mathrm{exch}}$	$E^{(2)}_{\mathrm{ind}}$	$E^{(2)}_{\mathrm{exch-ind}}$	$E^{(2)}_{\mathrm{disp}}$	$E^{(2)}_{\mathrm{exch-disp}}$	δ_{HF}	$E^{\mathrm{SAPT}+\delta_{\mathrm{HF}}}_{\mathrm{int}}$
Benzene-	T	−3.713	9.995	−3.192	2.958	−12.331	1.697	−0.760	−5.346
-Cyclopentane	Q	−3.709	9.986	−3.193	2.959	−12.539	1.752	−0.760	−5.503
Benzene-	T	−3.018	7.929	−2.479	2.238	−9.769	1.333	−0.560	−4.326
Neopentane	Q	−3.014	7.925	−2.478	2.238	−9.922	1.374	−0.551	−4.428
AcOH-	T	−2.984	8.304	−2.446	2.029	−9.738	1.121	−0.439	−4.152
-pentane	Q	−2.981	8.299	−2.448	2.031	−9.928	1.167	−0.442	−4.302
$AcNH_2$-	T	−3.993	10.293	−3.419	2.507	−11.407	1.463	−0.628	−5.183
-pentane	Q	−3.989	10.288	−3.422	2.509	−11.636	1.522	−0.631	−5.359
peptide-	T	−4.424	11.958	−3.583	2.808	−13.959	1.728	−0.678	−6.147
-pentane	Q	−4.417	11.947	−3.583	2.807	−14.219	1.794	−0.679	−6.350

Table S4: **Ground-state** interaction energy components (in milliHartree) at the **SAPT(CAS)** level of theory. aVXZ stands for aug-cc-pVXZ (X=D,T) basis sets of Dunning. All exchange energy terms given in the S^2 approximation. $E^{(2)}_{\mathrm{exch-ind}}$ and $E^{(2)}_{\mathrm{exch-ind}}$ in aVTZ are obtained via scaling, see Computational Details.

	X	$E^{(1)}_{\mathrm{elst}}$	$E^{(1)}_{\mathrm{exch}}$	$E^{(2)}_{\mathrm{ind}}$	$E^{(2)}_{\mathrm{exch-ind}}$	$E^{(2)}_{\mathrm{disp}}$	$E^{(2)}_{\mathrm{exch-disp}}$	δ_{HF}	$E^{\mathrm{SAPT}+\delta_{\mathrm{HF}}}_{\mathrm{int}}$
Benzene-	D	−3.329	8.549	−2.220	1.961	−10.686	1.305	−0.751	−4.419
-cyclopentane	T	−3.270	8.496	−2.305	2.036	−11.362	1.388	−0.760	−5.777
Benzene-	D	−2.546	6.536	−1.494	1.241	−8.397	0.985	−0.551	−3.675
-neopentane	T	−2.523	6.503	−1.599	1.327	−8.896	1.044	−0.560	−4.703
AcOH-	D	−2.426	6.614	−1.567	1.239	−8.071	0.769	−0.439	−3.442
-pentane	T	−2.451	6.693	−1.680	1.328	−8.843	0.843	−0.440	−4.549
AcNH2-	D	−3.371	8.389	−2.392	1.536	−9.606	1.052	−0.620	−4.391
-pentane	T	−3.337	8.351	−2.495	1.603	−10.379	1.197	−0.628	−5.689
peptide-	D	−3.708	9.723	−2.393	1.756	−11.905	1.262	−0.673	−5.265
-pentane	T	−3.682	9.674	−2.503	1.836	−12.798	1.357	−0.678	−6.793

Table S5: **Excited-state** interaction energy components (in milliHartree) at the **SAPT(CAS)** level of theory. aVXZ stands for aug-cc-pVXZ (X=D,T) basis sets of Dunning. All exchange energy terms given in the S^2 approximation. $E^{(2)}_{\mathrm{exch-ind}}$ and $E^{(2)}_{\mathrm{exch-ind}}$ in aVTZ are obtained via scaling, see Computational Details.

	X	$E^{(1)}_{\mathrm{elst}}$	$E^{(1)}_{\mathrm{exch}}$	$E^{(2)}_{\mathrm{ind}}$	$E^{(2)}_{\mathrm{exch-ind}}$	$E^{(2)}_{\mathrm{disp}}$	$E^{(2)}_{\mathrm{exch-disp}}$	δ_{CAS}	$E^{\mathrm{SAPT}+\delta_{\mathrm{CAS}}}_{\mathrm{int}}$
Benzene-	D	−3.115	8.259	−2.183	2.007	−10.329	1.226	−0.739	−4.873
-cyclopentane	T	−3.054	8.209	−2.260	2.078	−10.987	1.305	−0.746	−5.455
Benzene-	D	−2.306	6.202	−1.456	1.269	−8.088	0.910	−0.537	−4.005
-neopentane	T	−2.280	6.172	−1.551	1.352	−8.570	0.964	−0.543	−4.456
AcOH-	D	−2.493	6.624	−1.596	1.410	−8.300	0.825	−0.447	−3.977
-pentane	T	−2.433	6.562	−1.648	1.456	−8.940	0.889	−0.432	−4.546
AcNH2-	D	−3.538	9.141	−3.115	3.051	−9.858	1.202	−0.808	−3.924
-pentane	T	−3.484	9.074	−3.265	3.198	−10.614	1.294	−0.822	−4.619
peptide-	D	−3.669	9.827	−2.374	2.107	−12.042	1.306	−0.667	−5.512
-pentane	T	−3.611	9.741	−2.448	2.173	−12.915	1.400	−0.663	−6.322

Table S6: Total CASSCF energies (in Hartree) computed for the dimer and monomers in their ground and excited states in aug-cc-pVTZ.

	dimer	monomerA	monomerB
		ground state	
Benzene-Cyclopentan	−426.078 533	−230.852 756	−195.230 155
Benzene-Neopentane	−427.254 009	−196.404 550	−230.852 749
AcOH-Pentane	−424.433 227	−228.032 321	−196.404 380
AcNH2-Pentane	−404.530 470	−208.129 818	−196.404 349
Peptide-Pentane	−443.606 199	−196.404 355	−247.206 538
		excited state	
Benzene-Cyclopentan	−425.894 636	−230.668 890	−195.230 155
Benzene-Neopentane	−427.070 122	−196.404 550	−230.668 860
AcOH-Pentane	−424.175 736	−227.774 967	−196.404 380
AcNH2-Pentane	−404.308 504	−207.909 109	−196.404 349
Peptide-Pentane	−443.365 463	−246.966 172	−196.404 355

Table S7: Truncation errors of the dispersion energy computed with with $n = 4, \ldots, 20$ [see Eqs. (11) and (14) in the main text] with respect to the SAPT(CAS) reference [Eq.(1) in the main text] for the **A24 dataset**. "ref." denotes SAPT(CAS) values. MAE and MAX are mean absolute error and maximum error, respectively. Basis set is aug-cc-pVTZ. Energy unit is μE_h.

	4	6	8	10	12	20	ref
$H_2O \cdots H_2O$	1.6	−0.2	0.2	0.5	0.6	0.7	−4428
$H_2O \cdots NH_3$	2.2	0.2	0.6	0.8	0.9	1.0	−6229
$HCN \cdots HCN$	15.4	7.3	4.5	2.8	1.7	0.3	−2918
$HF \cdots HF$	4.8	0.1	−0.3	−0.2	−0.1	−0.1	−3142
$NH_3 \cdots NH_3$	1.6	0.7	0.8	0.9	1.0	1.0	−3899
$C_2H_2 \cdots C_2H_2$	0.8	1.4	1.0	0.7	0.5	0.3	−2295
$C_2H_4 \cdots C_2H_4$	−6.8	−2.3	−0.9	−0.4	−0.1	0.0	−3471
$CH_4 \cdots C_2H_4$	1.7	0.8	0.5	0.4	0.3	0.3	−1506
$H_2O \cdots C_2H_4$	0.2	−0.6	−0.2	0.1	0.2	0.3	−3734
$H_2O \cdots CH_4$	3.4	1.0	0.5	0.4	0.3	0.3	−1441
$HCOH \cdots C_2H_4$	1.7	3.4	3.7	3.5	3.1	1.7	−3570
$HCOH \cdots HCOH$	21.7	16.9	13.7	11.1	8.9	4.1	−7266
$HF \cdots CH_4$	6.0	1.3	0.3	0.0	0.0	−0.1	−2461
$NH_3 \cdots C_2H_4$	−1.4	−0.7	−0.1	0.1	0.2	0.3	−2699
$NH_3 \cdots CH_4$	3.3	1.3	0.7	0.4	0.4	0.3	−1821
$Ar \cdots C_2H_4$	0.1	−0.1	0.2	0.4	0.4	0.5	−1195
$BH_3 \cdots CH_4$	73.7	62.6	54.1	47.1	41.3	25.5	−4636
$C_2H_2 \cdots C_2H_2$	−0.1	0.7	0.4	0.2	0.1	0.0	−3348
$C_2H_4 \cdots C_2H_2$	−6.2	−1.8	−0.7	−0.3	−0.1	0.0	−3893
$C_2H_4 \cdots C_2H_4$	−14.6	−5.4	−2.3	−1.0	−0.4	0.0	−4507
$CH_4 \cdots C_2H_6$-1	5.1	1.9	1.4	0.8	0.5	0.3	−2727
$CH_4 \cdots C_2H_6$-2	3.3	1.4	0.5	0.1	−0.1	−0.2	−1995
$CH_4 \cdots CH_4$	2.8	1.0	0.3	−0.1	−0.2	−0.3	−1771
MAE	7.8	4.9	4.0	3.1	2.8	1.6	
MAX	73.7	62.6	54.1	47.1	41.3	25.5	

Table S8: Truncation errors of the dispersion energy computed with with $n = 6, 8, 10, 12, 14$ [see Eqs. (11) and (14) in the main text] with respect to the SAPT(CAS) reference [Eq. (1) in the main text] for the **TK21 dataset**. "ref." denotes SAPT(CAS) values. Basis set is aug-cc-pVTZ. Energy unit is μE_h.

	6	8	10	12	14	ref
$F^- \cdots HF$	−17.2	−12.5	−6.6	−3.3	−1.7	−22 754
$F^- \cdots H_2O$	−8.6	−6.8	−3.6	−1.7	−0.7	−14 237
$Na^+ \cdots H_2O$	−0.2	−0.1	0.0	0.0	0.0	−858
$HF \cdots HF$	0.2	−0.2	−0.1	0.0	0.0	−2959
$CH_4 \cdots CH_4$	1.0	0.3	0.0	−0.1	−0.2	−1525
$H_2O \cdots H_2O$	−0.9	−0.5	−0.2	−0.1	0.0	−4700
$NH_3 \cdots CH_4$	0.7	0.3	0.1	0.0	0.0	−1882
$NH_3 \cdots H_2O$	−0.8	−0.4	−0.2	−0.1	0.0	−6402
$N_2 \cdots N_2$	0.1	0.0	0.0	0.0	0.0	−728
$C_2H_2 \cdots C_2H_2$(PD)	1.4	1.0	0.6	0.4	0.3	−2278
$C_2H_2 \cdots C_2H_2$(S)	0.2	0.2	0.1	0.1	0.1	−1084
$C_2H_2 \cdots C_2H_2$(T)	1.2	0.8	0.6	0.4	0.3	−2219
$C_2H_6 \cdots HCN$	0.7	0.4	0.3	0.1	0.1	−1809
NCCN$\cdots$NCCN	−20.8	−14.1	−10.1	−7.4	−5.5	−3202
$P_2 \cdots P_2$	0.5	0.3	0.2	0.1	0.1	−3264
$N_2O \cdots$He(GM)	1.8	0.4	0.1	0.0	0.0	−508
$N_2O \cdots$He(LM)	1.0	0.2	0.1	0.0	0.0	−261
$CO_2 \cdots$He(GM)	1.6	0.4	0.1	0.0	0.0	−457
$CO_2 \cdots$He(LM)	0.8	0.2	0.1	0.0	0.0	−204
Ar$\cdots$Ar	−0.4	−0.1	0.0	0.0	0.0	−519
PCCP$\cdots$PCCP	1.7	2.8	1.9	1.2	0.7	−7913
MAE	3.0	2.0	1.2	0.7	0.5	
MAX	20.8	14.1	10.1	7.4	5.5	

References

(S1) Hapka, M.; Przybytek, M.; Pernal, K. Symmetry-Adapted Perturbation Theory Based on Multiconfigurational Wave Function Description of Monomers. *J. Chem. Theory Comput.* **2021**, *17*, 5538–5555.

(S2) Parrish, R. M.; Sherrill, C. D. Spatial assignment of symmetry adapted perturbation theory interaction energy components: The atomic SAPT partition. *J. Chem. Phys.* **2014**, *141*, 044115.

(S3) Řezáč, J.; Riley, K. E.; Hobza, P. S66: A Well-balanced Database of Benchmark Interaction Energies Relevant to Biomolecular Structures. *J. Chem. Theory Comput.* **2011**, *7*, 2427–2438.

(S4) Halkier, A.; Helgaker, T.; Jørgensen, P.; Klopper, W.; Koch, H.; Olsen, J.; Wilson, A. K. Basis-set convergence in correlated calculations on Ne, N_2, and H_2O. *Chem. Phys. Lett.* **1998**, *286*, 243–252.

(S5) Heßelmann, A.; Jansen, G.; Schütz, M. Density-functional theory-symmetry-adapted intermolecular perturbation theory with density fitting: A new efficient method to study intermolecular interaction energies. *J. Chem. Phys.* **2005**, *122*, 014103.
